# Model-Based Prediction of an Effective Adhesion Parameter Guiding Multi-Type Cell Segregation

**DOI:** 10.3390/e23111378

**Published:** 2021-10-21

**Authors:** Philipp Rossbach, Hans-Joachim Böhme, Steffen Lange, Anja Voss-Böhme

**Affiliations:** 1 DataMedAssist, HTW Dresden, 01062 Dresden, Germany; philipp.rossbach2@htw-dresden.de (P.R.); hans-joachim.boehme@htw-dresden.de (H.-J.B.); steffen.lange@htw-dresden.de (S.L.); 2 Faculty of Informatics/Mathematics, HTW Dresden—University of Applied Sciences, 01062 Dresden, Germany

**Keywords:** differential adhesion hypothesis, differential migration model, cell sorting, cellular automaton, statistical learning methods, high heterotypic interfacial tension hypothesis, pattern formation

## Abstract

The process of cell-sorting is essential for development and maintenance of tissues. Mathematical modeling can provide the means to analyze the consequences of different hypotheses about the underlying mechanisms. With the Differential Adhesion Hypothesis, Steinberg proposed that cell-sorting is determined by quantitative differences in cell-type-specific intercellular adhesion strengths. An implementation of the Differential Adhesion Hypothesis is the Differential Migration Model by Voss-Böhme and Deutsch. There, an effective adhesion parameter was derived analytically for systems with two cell types, which predicts the asymptotic sorting pattern. However, the existence and form of such a parameter for more than two cell types is unclear. Here, we generalize analytically the concept of an effective adhesion parameter to three and more cell types and demonstrate its existence numerically for three cell types based on in silico time-series data that is produced by a cellular-automaton implementation of the Differential Migration Model. Additionally, we classify the segregation behavior using statistical learning methods and show that the estimated effective adhesion parameter for three cell types matches our analytical prediction. Finally, we demonstrate that the effective adhesion parameter can resolve a recent dispute about the impact of interfacial adhesion, cortical tension and heterotypic repulsion on cell segregation.

## 1. Introduction

During the development of multicellular organisms, mechanical forces affect the internal states of cells as well as the interaction between cells, and they are, therefore, an integral part of all morphogenetic processes [1]. These forces are typically driven by molecular motors and transmitted via cytoskeleton elements and adhesion molecules within the cells and between them. Together, molecular motor driven movement and force transmission via adhesion complexes constitute two major self-organizing phenomena that drive tissue morphogenesis.

Since it constitutes a paradigmatic process for tissue morphogenesis, cell-sorting has found much attention both from experimental and theoretical research. It describes the tissue-scale segregation phenomenon that is observed when mixed heterotypic cell populations unmix into spatially confined homotypic cell clusters [2,3]. Over many decades this process has been studied in a number of in-vitro cell-sorting experiments for many different cell types [2,3,4,5,6,7]. To resolve which types of intercellular interaction guide individual mobile cells to find their homotypic neighbors, several theoretical hypotheses have been proposed and studied. Steinberg put forward the Differential Adhesion Hypothesis (DAH) which exploits the similarity of cell segregation with the unmixing of immiscible fluids such as water and oil. It states that cell-sorting is driven by the minimization of tissue surface tensions which result from quantitative differences in the strengths of cell-type specific intercellular adhesion [8]. However, Harris (1976) [9] remarked that tissue surface tension can result from several cellular mechanisms besides differential adhesion and suggested the differential contractility of cells as a major driver of cell-sorting. This is also acknowledged by the Differential Interfacial Tension Hypothesis (DITH) of Brodland and Chen (2000) [10]. There, the effects of intercellular adhesion and cellular contractility at cell–cell contacts are subsumed to the concept of differential interfacial tension at homo- and heterotypic cell contacts which determines the degree of mutual attachment between cells and thus drives cell-sorting. Canty et al. (2017) [11] demonstrated experimentally that repulsion at heterotypic cell–cell contacts with little to no contribution from adhesive or tensile differences between cell types can lead to cell-sorting and tissue separation. They propose the High Heterotypic Interfacial Tension Hypothesis (HIT) which asserts that heterotypic repulsion creates a situation where tension is strongly increased at heterotypic contacts compared to homotypic contacts and constitutes the major driver of cell-sorting and tissue separation.

The competing explanatory models DAH, DITH and HIT are unified within the Differential Migration Model (DMM) of Voss–Böhme and Deutsch (2010) [12]. They established that all the above hypotheses have in common that cell–cell contacts affect type-specifically the migration properties of the involved cells and parametrized this effect, independent of its specific nature - adhesion or repulsion, cortical tension or even signaling cascades. For the segregation of two cell types, the existence and form of an effective adhesion parameter (EAP) has been derived mathematically, which predicts the asymptotic sorting behavior [12].

However, while most previous studies focus on the simplest case of two cell types [7,13,14], the case of three and more cell types is more relevant in real biological systems. Studies of cell segregation and pattern formation with three or more cell types are still rare [15]. Although the DMM can be directly generalized to more than two cell types, it has only been studied for the minimal case of two cell types. In particular, the existence and form of the EAP for systems with three or more cell types remains an open question.

We study numerically the impact of the EAP and related parameters on the degree of tissue separation in terms of clear tissue boundaries as well as the separation time scale for mixtures of two cell types. We present new arguments to analytically predict the form of the EAP, thus generalizing the concept of the EAP to mixtures of arbitrary many cell types. We show numerically for three cell types that the analytically predicted EAP guides the asymptotic sorting behavior in the DMM, i.e., the EAP determines the asymptotic value of the normalized sum of heterotypic contacts. The form of the EAP on the asymptotic segregation behavior is independently quantified via two statistical learning methods, Support Vector Machines (SVM) and Logistic Regression model (Logit) [16]. The form estimated by the statistical learning methods matches our analytical prediction both for two and three cell types. Finally, we demonstrate that the EAP can explain different segregation behaviors in two cell- type Cellular Potts models which was previously attributed to the competing hypotheses DAH/DITH and HIT [11].

## 2. Materials and Methods

### 2.1. Differential Migration Model

**Figure 1:**
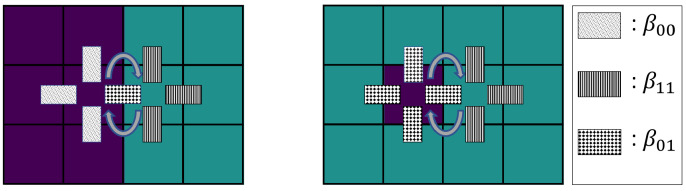
Example of a cell switch. (**a**) A cell switch at a straight interface between two clusters of type w=0 (purple) and w=1 (turquoise) has rate $c=\exp\left[-3\left(\beta_{00}+\beta_{11}\right)-2\beta_{01}\right]$ according to Equation (2). (**b**) A single cell of type w=0 inside a cluster of cells of type w=1 switches with rate $c=\exp\left[-3\beta_{11}-5\beta_{01}\right]$. The cell switch between two adjacent cells is highlighted (gray double arrows).

The DMM is a special probabilistic cellular automaton (PCA) based on Voss-Böhme and Deutsch [12], which has been analyzed for two cell types. Here, we present the key features of this model for the case of arbitrary many cell types:

*Lattice:* The PCA works on a squared lattice $S:={\left\{0,1,\cdots ,L\right\}}^{2}$ with periodic boundaries and we set L=25 throughout our numerical analysis.
*Cells:* To emulate biological cells, every lattice site x∈S is assigned a cell type w∈W of all possible cell types W:={0,1,⋯} in the system. In this way each cell regardless of its type, has approximately the same size and occupies exactly one lattice site. The number of cells of each type remains constant during the sorting process.
*Configurations:* A configuration $\eta_{t}$ of the lattice *S* represents the state of the model at time *t*. It belongs to the set of all possible configurations $X:=W^{S}=\left\{\eta :S\to W\right\}$.
*Migration:* A configuration η is changed by a cell position switch $\eta \to \eta_{xy}$ involving two adjacent lattice sites *x* and *y* with x,y∈S:
  (1) $$\eta_{xy}\left(z\right):=\left\{\begin{array}{c} \eta (z),\;z\neq x\;\mathrm{and}\;z\neq y\\ \eta (y),\;z=x\\ \eta (x),\;z=y\;. \end{array}\right.$$
  Position switches between two cells of the same type do not change the configuration and are therefore neglected. Thus, neighboring cells situated at lattice sites *x* and *y* only switch their positions if they are heterotypic, i.e., η(x)≠η(y).
*Differential adhesion:* We assume that stronger bonds to neighboring cells hinder cell motility. Accordingly, a cell switch $\eta \to \eta_{xy}$ occurs with rate c(x,y,η). The rate c(x,y,η) depends on the parameters $\beta_{\eta (x)\eta (z)}$ and $\beta_{\eta (y)\eta (z)}$, which represent the binding strengths between cells at lattice sites *x* and *y* to positions from the von-Neumann-1 neighborhoods N(x) and N(y), see Figure 1. The von-Neumann-1 neighborhood of a lattice site corresponds to all neighboring lattice sites with Manhattan distance one. The cell switch rate of the two adjacent lattice sites x,y∈S with x∈N(y) and y∈N(x) is as follows:
  (2) $$c\left(x,y,\eta \right):=\left\{\begin{array}{cc} \exp\left(-\sum_{z\in N(x)}\beta_{\eta (x)\eta (z)}-\sum_{z\in N(y)}\beta_{\eta (y)\eta (z)}\right),\; & \eta (x)\neq \eta (y)\\ 0,\; & \mathrm{otherwise}\;. \end{array}\right.$$

Notice that the particular mechanism which affects the cellular motility on intercellular contact is not specified. The parameters $\beta_{ij}$ can be interpreted as repulsion, if $\beta_{ij}<0$, enhancing cellular motility, and it can be interpreted as binding strength resulting from the interplay of adhesion and relaxed cortical tension at cell–cell contact, if $\beta_{ij}>0$, inhibiting cellular motility. The details on the numerical implementation of this PCA are elaborated in the Appendix A. Depending on how many cell types are considered the number of intercellular adhesion parameters varies. For this, the vector $\beta_{\left(N\right)}$ of all intercellular adhesion parameters occurring in a system with *N* cell types is introduced, for instance

$$\begin{array}{c} \beta_{\left(N=2\right)}:={\left(\beta_{00},\beta_{11},\beta_{01}\right)}^{T}\;\mathrm{for}\;2-\mathrm{cell}-\mathrm{type}\;\mathrm{systems}\;\mathrm{and}\;\\ \beta_{\left(N=3\right)}:={\left(\beta_{00},\beta_{11},\beta_{22},\beta_{01},\beta_{02},\beta_{12}\right)}^{T}\;\mathrm{for}\;3-\mathrm{cell}-\mathrm{type}\;\mathrm{systems}.\; \end{array}$$

For a 2-cell-type model, an effective adhesion parameter (EAP)

$$\beta_{\left(N=2\right)}^{*}:=\beta_{00}+\beta_{11}-2\beta_{01}$$

was analytically predicted in Voss-Böhme and Deutsch [12]. The EAP determines the asymptotic cell segregation behavior of systems with N=2 cell types regardless of the value combinations of $\beta_{\left(2\right)}$. The higher the EAP the more segregated the cell types become.

For simulation of the DMM model a Gillespie-related algorithm is used where independent cell switches, see Equation (1), are chosen and the waiting times between these events are calculated based on the cell switch rates, see Equation (2), within lattice configuration η. For details about this algorithm, see Appendix A, Algorithm A1. Each numerical simulation yields a time-series of lattice configurations $\eta_{t}$, since every cell switch, i.e., whenever two neighboring cells switch their position, see Equation (1), results in a new configuration. The variable *t* refers to the number of cell switches performed.

### 2.2. Order Indicator

To quantify the level of cell segregation an order indicator is introduced that quantifies the level of cell segregation of a lattice configuration η. The order indicator ω(η) is the normalized sum of homotypic cell–cell contacts within a configuration η. This indicator increases the more cells of the same type are clustered together. In detail, the set of all homotypic von-Neumann-1 neighbors of a cell of type η(x) at position x∈S is given by

H(x,η):={z∈S∣η(x)=η(z),z∈N(x)}.

With $n_{=}\left(x,\eta \right):=\left|H\left(x,\eta \right)\right|$, the total sum of homotypic connections is defined as

(3) $$d\left(\eta \right):=\frac{\sum_{x\in S}n_{=}\left(x,\eta \right)}{2}\;.\;$$

The highest amount $d_{max}$ and the lowest amount $d_{min}$ of homotypic lattice site connections are reached in the case of a fully sorted and chessboard-patterned configuration, respectively. The exact values for 2- and 3-cell-type systems are listed in Appendix B. With $d_{max}$ and $d_{min}$, the order indicator value ω(η) for configuration η is defined as the normalization of d(η):

$$\omega \left(\eta \right):=\frac{d\left(\eta \right)-d_{min}}{d_{max}-d_{min}}$$

**Figure 2:**
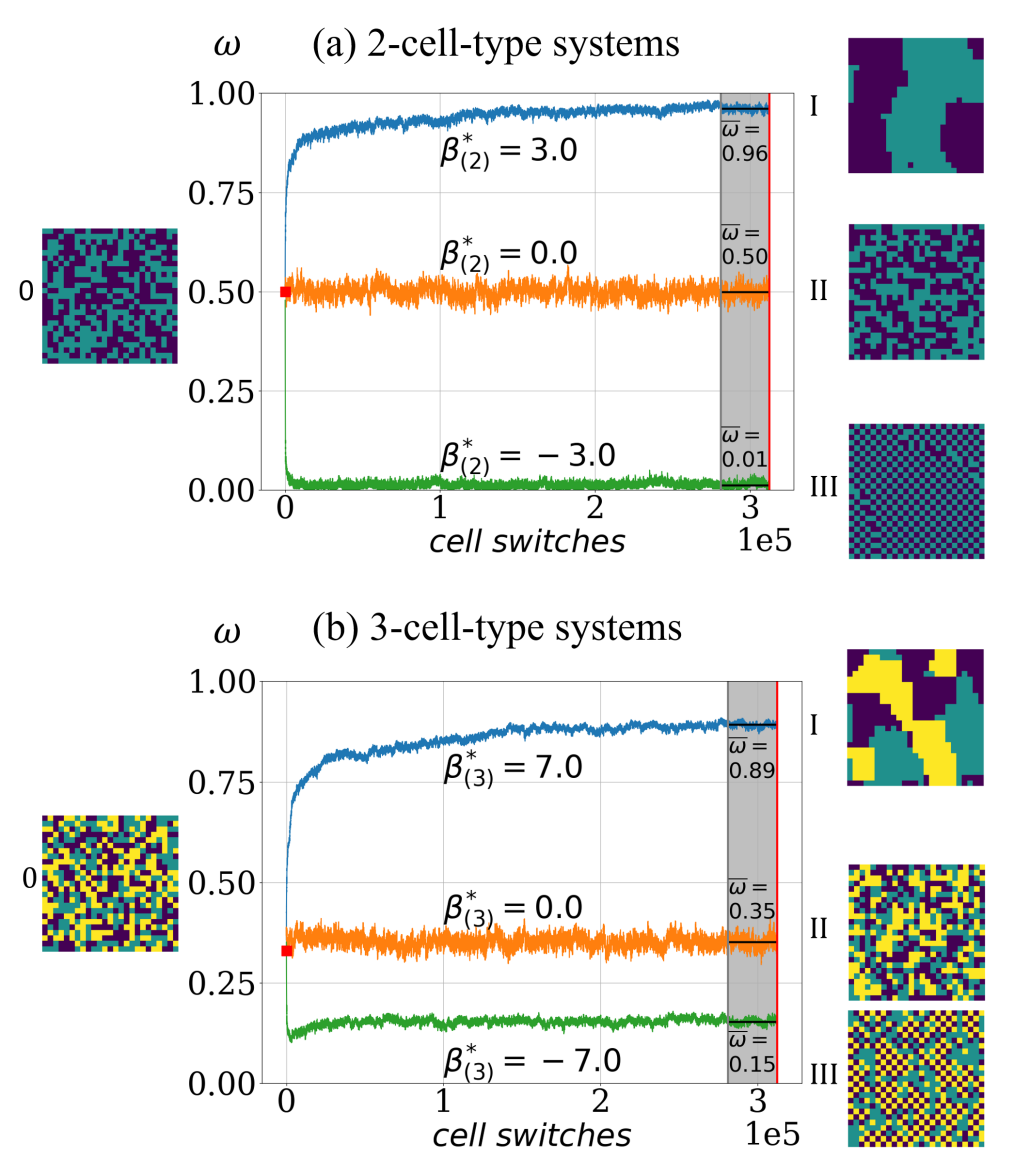
Illustration of the principal cell-sorting behavior in the model. Depending on the effective adhesion parameter the model exhibits asymptotically one of three basic cell-sorting states. (**a**) Simulated order indicator ω(t) in the case of 2-cell-type system for three different sets of intercellular adhesion parameters $\beta_{\left(2\right)}$: (1.2,−0.2,−1.0) (blue), (1.1,−1.1,0.0) (orange), (−1.3,0.3,1.0) (green) for three different cell-type systems, each corresponding to a distinct asymptotic segregation: a cell population with sorted patterns as in configuration I (blue), a not fully cell-type segregated population as in II (orange) and a cell type mixture population with chessboard pattern as in III (green). Configuration 0 illustrates the random start configuration $\eta_{t=0}$ with an initial order indicator value ω(t=0)≈0.5 (marked as red square at t=0). The simulation termination condition is t=312499 cell switches (red vertical line). The asymptotic value $\bar{\omega }$ (indicated for each case as black horizontal line at the end) is computed as the average over the last 10% of cell switches (gray area). (**b**) Analogous for examples of 3-cell-type systems, starting with random configuration, i.e., initial order indicator value ω(t=0)≈0.33. The three different sets of intercellular adhesion parameters $\beta_{\left(3\right)}$ are: (0.6,0.6,2.3,−0.9,−1.1,−1.5) (blue), (−0.8,−0.1,1.0,−0.1,−0.2,0.4) (orange) and (−0.6,−0.6,−2.3,0.6,2.4,0.5) (green).

Thus, ω(η) takes values in [0,1], where configurations with almost perfect sorted patterns, such as in Figure 2I, yield ω(η) values above 0.95. In the case of chessboard configurations as in Figure 2III, the order indicator values are below 0.05. For a random lattice configuration, the order indicator value is ω(η)≈0.5, in the case of a 2-cell-type system, and it is ω(η)≈0.33 for a 3-cell-type system.

With the order indicator ω(η), a time-series of configurations $\eta_{t}$ can be converted into a time-series of order indicator values $\omega (\eta_{t})$, in short ω(t). Furthermore, the asymptotic cell segregation level for a simulation is estimated as the average over the last 10% of the time-series ω(t) and is denoted $\bar{\omega }$, see Figure 2 for an illustration.

## 3. Results

### 3.1. Cell System Parameters for Two Cell Types

We present a new simple argument for the form of the effective adhesion parameter $\beta_{\left(2\right)}^{*}$ guiding the asymptotic behavior in the DMM for two cell types [12] to generalize it to systems with arbitrary many cell types. For this, we introduce two additional system parameters $\beta_{\left(2\right)}^{s}$ and $\beta_{\left(2\right)}^{\Delta }$ besides $\beta_{\left(2\right)}^{*}$, which determine how changes to the intercellular adhesion parameters $\beta_{\left(2\right)}$ affect the asymptotic cell segregation behavior as well as the dynamics. Although the parameters $\beta_{\left(2\right)}^{s}$ and $\beta_{\left(2\right)}^{*}$ set the temporal scale of the waiting times and the asymptotic level of segregation, respectively, we argue that the parameter $\beta_{\left(2\right)}^{\Delta }$ determines the number of cell switches required to reach the asymptotic level of segregation.

Indeed, the addition of a constant θ to all intercellular adhesion parameters simultaneously like

(4) $$\beta_{\left(2\right)}^{{}^{\prime }}=\beta_{\left(2\right)}+\theta a_{s}\;\mathrm{with}\;a_{s}={\left(1,1,1\right)}^{T}$$

rescales the cell switch rates $c_{\beta }\left(x,y,\eta \right)$ in the DMM by a factor exp(−8θ), since

(5) $$\begin{array}{cc} c_{\beta_{\left(2\right)}^{{}^{\prime }}}\left(x,y,\eta \right) & =\exp\left(-\sum_{z\in N(x)}\left(\beta_{\eta (x)\eta (z)}+\theta \right)-\sum_{z\in N(y)}\left(\beta_{\eta (y)\eta (z)}+\theta \right)\right)\\ \; & =\exp\left(-8\theta \right)\cdot c_{\beta_{\left(2\right)}}\left(x,y,\eta \right)\;,\; \end{array}$$

independent of the actual configuration η∈X and the considered lattice sites x,y∈S. This factor does not alter the relation between the cell switch rates, but only rescales all waiting times between cell switches by exp(−8θ). Thus, the spatio-temporal order of the cell sorting dynamics and the asymptotic behavior are not affected. In order to parametrize this rescaling, a scaling parameter $\beta_{\left(2\right)}^{s}$

(6) $$\beta_{\left(2\right)}^{s}=\beta_{00}+\beta_{11}+\beta_{01}=\langle \beta_{\left(2\right)},a_{s}\rangle$$

is introduced, where the functional, which maps $\beta_{\left(2\right)}\to \beta_{\left(2\right)}^{s}$, is given as a scalar product with the vector $a_{s}={\left(1,1,1\right)}^{T}$.

Secondly, we notice that the choice which cell type is denoted by type w=0 and which one by w=1 is arbitrary and the dynamics in the model is invariant against relabeling of the cell types. This invariance is reflected in the cell switch rates c(x,y,η) as well as in the order indicator ω, which quantifies the level of segregation of a configuration η as sum of homotypic lattice site connections. Accordingly, the functional which maps the intercellular adhesion parameters $\beta_{\left(2\right)}$ to the effective adhesion parameter $\beta_{\left(2\right)}^{*}$ must reflect this invariance as well. This implies that an effective adhesion parameter $\beta_{\left(2\right)}^{*}$, which controls the asymptotic sorting behavior, assuming it exists and is determined by a linear functional on the intercellular adhesion parameters, has the form

(7) $$\begin{array}{cc} \beta_{\left(2\right)}^{*} & =a\left(\beta_{00}+\beta_{11}\right)+b\beta_{01}=\langle \beta_{\left(2\right)},a_{*}\rangle \end{array}$$

with two real-valued constants a, b and a vector $a_{*}={\left(a,a,b\right)}^{T}$ introduced analogously to Equation (6) to express the functional. The constants a, b are set by the condition that the asymptotic level of segregation and thus the differential adhesion parameter $\beta_{\left(2\right)}^{*}$ must not be affected by the temporal scaling parameter $\beta_{\left(2\right)}^{s}$, which implies

(8) $$\langle a_{*},a_{s}\rangle =0\;\Rightarrow \;2a=-b\;.$$

Without loss of generality, we can neglect an additional offset and factor in Equation (7) and choose a=1 for which follows

(9) $$\begin{array}{cc} \beta_{\left(2\right)}^{*} & =\beta_{00}+\beta_{11}-2\beta_{01}=\langle \beta_{\left(2\right)},a_{*}\rangle \\ a_{*} & ={\left(1,1,-2\right)}^{T}\;. \end{array}$$

We present an additional heuristic argument to make the assumption of a linear dependency in Equation (7) plausible and to estimate that the critical value of the effective adhesion parameter, at which the system remains randomly mixed, should be zero: Consider two limit scenarios of a cell switch, first two cells at a straight interface between clusters and secondly a single cell of one type moving inside a cluster of opposite type. As illustrated in Figure 1a, two cells of different type at the interface switch their position and thus cause a less segregated configuration with a rate $c_{\mathrm{mix}}=\exp\left[-3\left(\beta_{00}+\beta_{11}\right)-2\beta_{01}\right]$. In contrast, a cell of type w=0 inside a cluster of type w=1 switches position with a given neighbor with rate $c_{\mathrm{unmix},\;0}=\exp\left[-3\beta_{11}-5\beta_{01}\right]$, see Figure 1b. Analogously, for a single cell of type w=1 in a cluster of type w=1 the rate is $c_{\mathrm{unmix},\;1}=\exp\left[-3\beta_{00}-5\beta_{01}\right]$. Both types of switches are necessary to move a single cell of opposite type out of a cluster. Since the former switch with rate $c_{\mathrm{mix}}$ reduces the level of segregation while the latter two switches with rates $c_{\mathrm{unmix},\;0}$, $c_{\mathrm{unmix},\;1}$ are required to increase segregation, the ratio between these types of switches $c_{\mathrm{unmix},\;0}/c_{\mathrm{mix}}\cdot c_{\mathrm{unmix},\;1}/c_{\mathrm{mix}}=\exp\left[3\left(\beta_{00}+\beta_{11}-2\beta_{01}\right)\right]$ should determine the level of the asymptotic segregation. In particular, the point at which the system remains mixed is expected where mixing and unmixing rates are equal, i.e., where the ratio of the rates becomes 1. Since the exponential function is monotonous, it is sufficient to focus on the exponent, which is a multiple of the effective adhesion parameter $\beta_{\left(2\right)}^{*}=\beta_{00}+\beta_{11}-2\beta_{01}$. Thus, the critical value 1 of the ratio of rates translates into a critical parameter $\beta_{\left(2\right)}^{*}=0$ for which a two-cell-type system is expected to remain mixed.

After establishing the system parameters $\beta_{\left(2\right)}^{s}$ and $\beta_{\left(2\right)}^{*}$, which are defined via scalar products with the corresponding vectors $a_{s}$, $a_{*}$, we point out that there is a third system parameter $\beta_{\left(2\right)}^{\Delta }$ defined by the vector perpendicular to $a_{s}$ and $a_{*}$

(10) $$\begin{array}{cc} \beta_{\left(2\right)}^{\Delta } & =|\beta_{00}-\beta_{11}\left|=\right|\langle \beta_{\left(2\right)},a_{\Delta }\rangle |\\ a_{\Delta } & ={\left(1,-1,0\right)}^{T}∼a_{s}\times a_{*}\;. \end{array}$$

**Figure 3:**
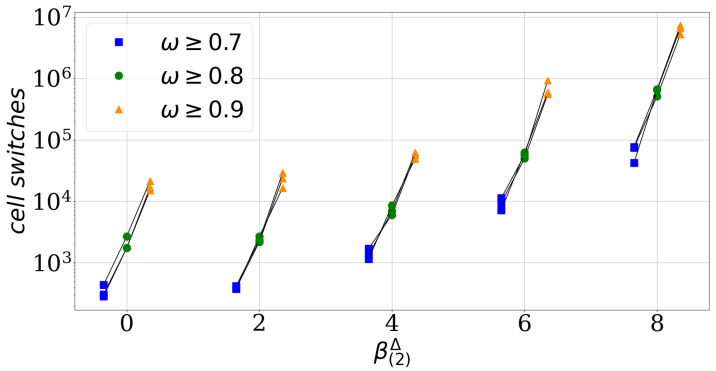
The convergence parameter slows down the sorting process. For each $\beta_{\left(2\right)}^{\Delta }$ value a different intercellular adhesion parameter $\beta_{\left(2\right)}$ with $\beta_{\left(2\right)}^{*}=3.0$ is used and every simulation is repeated three times, see black lines. When a simulation reached an order indicator value ω≥0.7, ≥0.8 and ≥0.9 for the first time the corresponding cell switch number is marked with a blue square, green dot and orange triangle. Each simulation has a random start configuration $\eta_{t=0}$ with an initial order indicator value $\omega (\eta_{t=0})\approx 0.5$ and the intercellular adhesion parameters for each convergence speed parameter $\beta_{\left(2\right)}^{\Delta }$ are: (0.5,0.5,−1) for 0, (−0.5,1.5,−1) for 2, (−1.5,2.5,−1) for 4, (3.5,−2.5,−1) for 6 and (−3.5,4.5,−1) for 8.The convergence speed parameter.

The parameter $\beta_{\left(2\right)}^{\Delta }$ quantifies the difference between the homotypic adhesion strengths of the two cell types. It becomes zero if both types have the same strength of homotypic adhesion. We use the absolute value in the definition of the parameter $\beta_{\left(2\right)}^{\Delta }$ as we are not interested in which cell type has the stronger homotypic adhesion. We find numerically that the parameter $\beta_{\left(2\right)}^{\Delta }$ affects how many cell switches are required to reach the asymptotic level of segregation, see Figure 3. For a fixed effective adhesion parameter $\beta_{\left(2\right)}^{*}=3$, which means for the same asymptotic level of cell segregation, Figure 3 shows the number of cell switches required to reach certain thresholds of cell segregation, here ω≥0.7, ≥0.8 and ≥0.9. This number increases with increasing $\beta_{\left(2\right)}^{\Delta }$ independently of the chosen thresholds.

This numerical observation is supported by a heuristic argument: Consider again the limit scenarios of a cell switch, i.e., two cells at a straight interface between clusters which switches with a particular neighbor with rate $c_{\mathrm{mix}}=\exp\left[-3\left(\beta_{00}+\beta_{11}\right)-2\beta_{01}\right]$ and a single cell of one type within a cluster of opposite type which switches with rate $c_{\mathrm{unmix},\;0}=\exp\left[-3\beta_{11}-5\beta_{01}\right]$, or $c_{\mathrm{unmix},\;1}=\exp\left[-3\beta_{00}-5\beta_{01}\right]$ respectively. In the symmetric case of equal homotypic adhesion parameters $\beta_{00}=\beta_{11}$, which implies $\beta_{\left(2\right)}^{\Delta }=0$ and $c_{\mathrm{unmix},\;0}=c_{\mathrm{unmix},\;1}$, cell switches of the two single cells in either cluster are equally frequent and these switches occur in a certain ratio with cell switches at the interface. If the symmetry is broken by increasing $\beta_{\left(2\right)}^{\Delta }$ at constant $\beta_{\left(2\right)}^{*}$ and $\beta_{\left(2\right)}^{s}$, for instance $\beta_{00}>\beta_{11}$, implying that $c_{\mathrm{unmix},\;0}>c_{\mathrm{unmix},\;1}$, the single cell of type w=0 is favored to leave the cluster of opposite type in comparison to the symmetric case and in particular compared to the other single cell of type w=1. Thus, segregation is now limited by the slower process of the single cell of type w=1 moving out of clusters of type w=0. Moreover, the rate $c_{\mathrm{mix}}$ of a cell switch at the interface is constant if only the parameter $\beta_{\left(2\right)}^{\Delta }$ is altered. Thus, in between two switches of a single cell of type w=1 (rate $c_{\mathrm{unmix},\;1}$) out of a type w=0 cluster there are more cell switches at the interface (rate $c_{\mathrm{mix}}$) than in the symmetric case. Please note that these back-and-forth switches at the interface do not progress the segregation but increase the number of total cell switches. This scenario illustrates how an increase of the parameter $\beta_{\left(2\right)}^{\Delta }$ increases the number of cell switches required to reach the asymptotic level of segregation. Thus, we denote $\beta_{\left(2\right)}^{\Delta }$ as convergence speed parameter.

Please note that the convergence speed parameter $\beta_{\left(2\right)}^{\Delta }$ in Equation (10) is proportional to the Euclidean distance between $\beta_{\left(2\right)}$ and the related symmetric adhesion parameters $\beta_{\left(2\right)}^{\mathrm{sym}}$ with the same values of $\beta_{\left(2\right)}^{*}$ and $\beta_{\left(2\right)}^{s}$. Indeed, for a given vector of adhesion parameters $\beta_{\left(2\right)}={\left(\beta_{00},\beta_{11},\beta_{01}\right)}^{T}$ with system parameters $\beta_{\left(2\right)}^{s}$, $\beta_{\left(2\right)}^{*}$, and $\beta_{\left(2\right)}^{\Delta }$, the corresponding symmetric adhesion parameters $\beta_{\left(2\right)}^{\mathrm{sym}}=\left(\frac{1}{2}\left[\beta_{00}+\beta_{11}\right],\frac{1}{2}\left[\beta_{00}+\beta_{11}\right],\beta_{01}\right)$ have the same system parameters $\beta_{\left(2\right)}^{s}$ and $\beta_{\left(2\right)}^{*}$ but a convergence speed parameter of zero. The Euclid distance of the adhesion parameters $\beta_{\left(2\right)}$ to this symmetric case $\beta_{\left(2\right)}^{\mathrm{sym}}$ is

(11) $$∥\beta_{\left(2\right)}-\beta_{\left(2\right)}^{\mathrm{sym}}∥=\sqrt{\frac{1}{2}}\left|\beta_{00}-\beta_{11}\right|∼\beta_{\left(2\right)}^{\Delta }\;.$$

Since the convergence speed parameter is zero, the symmetric case $\beta_{\left(2\right)}^{\mathrm{sym}}$ leads to the fastest convergence to the asymptotic level of segregation for the given system parameters $\beta_{\left(2\right)}^{s}$, $\beta_{\left(2\right)}^{*}$. In contrast to the definition of the convergence speed parameter $\beta_{\left(2\right)}^{\Delta }$ in Equation (10), the definition of $\beta_{\left(2\right)}^{\Delta }$ via Equation (11) can be generalized to *N* cell types, see below.

### 3.2. The Effective Adhesion Parameter for Arbitrary Number of Cell Types

For a mix of more than two cell types neither the form nor the existence of an effective adhesion parameter are known. Assuming that for an arbitrary number *N* of cell types an effective adhesion parameter $\beta_{\left(N\right)}^{*}$ exists, we postulate its form based on the generalization of our arguments for the case of two cell types. We also postulate the form of the convergence speed parameter $\beta_{\left(N\right)}^{\Delta }$ as introduced for two cell types.

The scaling parameter is directly generalized from Equation (6) to *N* cell types

(12) $$\begin{array}{cc} \beta_{\left(N\right)}^{s} & =\sum_{i=0}^{N-1}\beta_{ii}+\sum_{i<j}^{N-1}\beta_{ij}=\langle \beta_{\left(N\right)},a_{s}\rangle \\ a_{s} & ={\left(1,\ldots ,1\right)}^{T}\;. \end{array}$$

and only scales all waiting times between cell switches as in the case of two cell types.

The invariance against relabeling of the cell types applies as well to *N* cell types which implies, analogously to Equation (7),

(13) $$\begin{array}{cc} \beta_{\left(N\right)}^{*} & =a\sum_{i=0}^{N-1}\beta_{ii}+b\sum_{i<j}^{N-1}\beta_{ij}=\langle \beta_{\left(N\right)},a_{*}\rangle \end{array}$$

where the constants a, b are set again by the condition

(14) $$\langle a_{*},a_{s}\rangle =0\;\Rightarrow \;Na=-\frac{N(N-1)}{2}b\;.$$

Choosing again a=1, the effective adhesion parameter has the form

(15) $$\beta_{\left(N\right)}^{*}=\sum_{i=0}^{N-1}\beta_{ii}-\frac{2}{\left(N-1\right)}\sum_{i<j}^{N-1}\beta_{ij}$$

with corresponding vector $a_{*}$. For N=2 this equals the result for two cell types from Equation (9) and for N=3 this takes the form

(16) $$\beta_{\left(3\right)}^{*}=\beta_{00}+\beta_{11}+\beta_{22}-\left(\beta_{01}+\beta_{02}+\beta_{12}\right)\;.$$

In contrast to the case of two cell types, the space perpendicular to the vectors $a_{s}$ and $a_{*}$ for N>2 is not one- but [N+N(N−1)/2−2]-dimensional, i.e., two-dimensional for N=3. Thus, the definition of a convergence speed parameter $\beta_{\left(N\right)}^{\Delta }$, analogous to Equation (10), is ambiguous. One option is to consider all pairs of cell types and their corresponding convergence speed parameters, e.g., $|\beta_{00}-\beta_{11}|$, $|\beta_{00}-\beta_{22}|$, and $|\beta_{11}-\beta_{22}|$ for N=3. Since the segregation of all cell types requires the segregation of each pair of cell types, these pair-wise parameters should predict the convergence speed on subsets of intercellular adhesion parameters analogously to Figure 3. For instance, when two vectors of intercellular adhesion parameters are equal except they differ in one of these pair-wise convergence speed parameters, the one with the smaller parameter leads to a faster convergence. However, these pair-wise parameters do not allow comparison if several of them differ between two vectors of adhesion parameters and the influence of the heterotypic parameters is not even considered. Thus, the convergence speed parameter $\beta_{\left(N\right)}^{\Delta }$ is instead defined by the generalization of Equation (11)

(17) $$\begin{array}{cc} \beta_{\left(N\right)}^{\Delta } & =∥\beta_{\left(N\right)}-\beta_{\left(N\right)}^{\mathrm{sym}}∥\\ \beta_{\left(N\right)}^{\mathrm{sym}} & =\left({\bar{\beta }}_{\mathrm{hom}},\ldots ,{\bar{\beta }}_{\mathrm{hom}},{\bar{\beta }}_{\mathrm{het}},\ldots ,{\bar{\beta }}_{\mathrm{het}}\right)\\ {\bar{\beta }}_{\mathrm{hom}} & =\frac{1}{N}\sum_{i=0}^{N-1}\beta_{ii}\;,\;{\bar{\beta }}_{\mathrm{het}}=\frac{2}{N(N-1)}\sum_{i<j}^{N-1}\beta_{ij}\;. \end{array}$$

We postulate, in analogy to the case of two cell types, that the parameter $\beta_{\left(N\right)}^{\Delta }$ determines the number of cell switches required to reach the asymptotic level of segregation for a given effective adhesion parameter $\beta_{\left(N\right)}^{*}$.

### 3.3. Numerical Evidence for the Effective Adhesion Parameter

We test our analytical prediction for the effective adhesion parameter for more than two cell types, by comparison with numerical simulations of the DMM model for two and three cell types. We find in both cases that the proposed effective adhesion parameter determines the asymptotic level of cell segregation. However, there are deviations due to the limited simulation time, from which the asymptotic level of the order indicator $\bar{\omega }$ is extrapolated. This is revealed by additionally linking the simulation results to the convergence speed parameter $\beta^{\Delta }$, introduced above. For two cell types, we observe that for small values of $\beta_{\left(2\right)}^{\Delta }$, which refers to simulations which converge quickly to their asymptotic value, the dependency between $\beta_{\left(2\right)}^{*}$ and $\bar{\omega }$ agrees almost perfectly with the analytically proven prediction. Deviations from the analytical prediction are the most pronounced the larger the convergence speed parameter is. For three cell types, we observe an analogous relation between deviations from the postulated dependency on the effective adhesion parameter $\beta_{\left(3\right)}^{*}$ and a convergence speed parameter $\beta_{\left(3\right)}^{\Delta }$ as introduced above.

#### 3.3.1. The Asymptotic Level of Cell Segregation Depends on the Effective Adhesion Parameter

**Figure 4:**
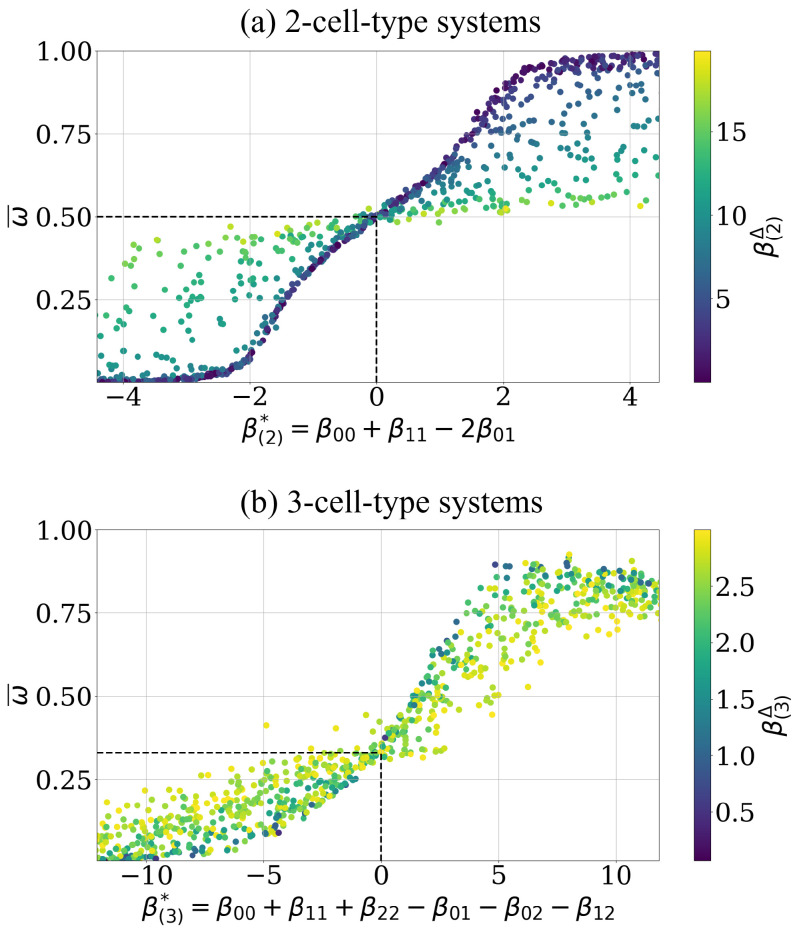
The level of the asymptotic cell segregation is predicted by the effective adhesion parameter. (**a**) Each point corresponds to a simulation whose intercellular adhesion parameter is drawn uniform, i.e., $\beta_{\left(2\right)}∼U{\left[-10,10\right]}^{3}$, with a random start configuration, i.e., initial order indicator ω(t=0)≈0.5. The curve $\omega (\beta_{\left(2\right)}^{*})$ intersects with the predicted point $\omega (\beta_{\left(2\right)}^{*}=0)=0.5$ (highlighted by black dashed lines). A data point represents the asymptotic estimate $\bar{\omega }$ of this simulation after ≈300,000 cell switches, along with the corresponding effective adhesion parameter $\beta_{\left(2\right)}^{*}$ and convergence parameter $\beta_{\left(2\right)}^{\Delta }$ (color bar). The corresponding shift parameter $\beta_{\left(2\right)}^{s}$ is not shown. (**b**) Analogous representation for the data points of 3-cell-type systems starting with random configurations, i.e., initial order indicator values ω(t=0)≈0.33. The curve $\omega (\beta_{\left(3\right)}^{*})$ intersects with the predicted point $\omega (\beta_{\left(3\right)}^{*}=0)=0.33$ (highlighted by black dashed lines). The intercellular adhesion parameters are also drawn uniform, i.e., $\beta_{\left(3\right)}∼U{\left[-10,10\right]}^{6}$ and have an additional condition such as $\beta_{\left(3\right)}^{\Delta }<3$, see Equation (17), to reduce scattering for visibility, see text for details.

At first the dependence of the level of asymptotic cell segregation $\bar{\omega }$ on the effective adhesion parameter $\beta^{*}$ is tested. For the 2-cell-type, the effective adhesion parameter clearly determines the asymptotic sorting state, see dark blue points in Figure 4a, i.e., $\bar{\omega }$ increases with increasing $\beta_{\left(2\right)}^{*}$, as predicted by Voss-Böhme and Deutsch [12], and all configurations stay randomly mixed at $\beta_{\left(2\right)}^{*}=0$, i.e., $\bar{\omega }\left(\beta^{*}=0\right)=0.5$ (see black dashed lines), as predicted by the heuristic argument above. The monotonic dependence of the asymptotic order parameter $\bar{\omega }$ on the proposed effective parameter $\beta_{\left(2\right)}^{*}$ is supported by a value of 0.81 of the spearman rank correlation coefficient (Kendall rank correlation coefficient of 0.58). Additionally, Figure 4a shows the asserted influence of the convergence speed parameter $\beta_{\left(2\right)}^{\Delta }$ on level of cell segregation dynamics. More precisely, the higher the $\beta_{\left(2\right)}^{\Delta }$ value is the slower is the sorting process and the closer the respective $\bar{\omega }$ value remains to ω(t=0)≈0.5 until the end of the simulation. For these intercellular adhesion parameters $\beta_{\left(2\right)}$, the asymptotic value of the order indicator has not yet been reached, which is why they are scattered above and below the increasing line, indicated by the dark blue points in Figure 4a. Note, that also the effective adhesion parameter $\beta^{*}$ shows impact on the amount of cell switches needed for a system to reach its asymptotic cell segregation $\bar{\omega }$. This is further investigated in Appendix D.

Analogous results for the case of three cell types with the postulated effective adhesion parameter are shown in Figure 4b. Qualitatively the same functional relation between the hypothesized effective adhesion parameter $\beta_{\left(3\right)}^{*}$ and $\bar{\omega }$ is visible as in the case of two cell types, see dark blue points in Figure 4b. This includes the prediction that all configurations stay randomly mixed at $\beta_{\left(3\right)}^{*}=0$, i.e., $\bar{\omega }\left(\beta^{*}=0\right)=0.33$ (see black dashed lines). The monotonic dependence of the asymptotic order parameter $\bar{\omega }$ on the proposed effective parameter $\beta_{\left(3\right)}^{*}$ is supported by a value of 0.79 of the Spearman rank correlation coefficient (Kendall rank correlation coefficient of 0.53), which is of similar quality as in the case of two cell types. Based on the analogy of the numerical results for two and three cell types, we conclude that $\beta_{\left(3\right)}^{*}$ predicts the level of sorting reached asymptotically such as $\beta_{\left(2\right)}^{*}$ does in the 2-cell-type case. Correspondingly, the asymptotic cell segregation exhibits the postulated influence of the convergence speed parameter $\beta_{\left(3\right)}^{\Delta }$. Note that the restriction in Figure 4b to simulations with an $\beta_{\left(3\right)}^{\Delta }<3.0$ ensures a sufficient sampling of data points with smaller $\beta_{\left(3\right)}^{\Delta }$ parameter values. Smaller $\beta^{\Delta }$ values result in more simulations reaching their asymptotic cell segregation.

**Figure 5:**
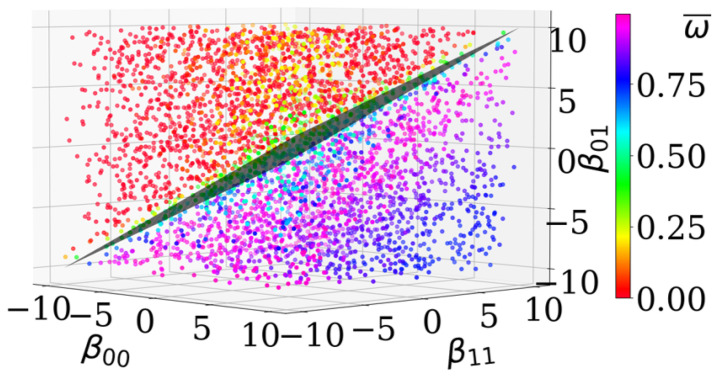
A plane with the analytically derived normal vector separates data points respective to their estimated asymptotic level of cell segregation. The points refer to the same data as in Figure 4a. The coloration represents the level of asymptotic cell segregation $\bar{\omega }$. The analytically predicted plane (gray rhomb in the middle) with the normal vector $a_{*}$ is $E:\beta_{00}+\beta_{11}-2\beta_{01}=\langle \beta_{\left(2\right)},a_{*}\rangle =0$.

For the 2-cell-type system, we can additionally visualize the effective adhesion parameter $\beta_{\left(2\right)}^{*}$ as a plane in 3D, see Figure 5, with $E:\left(\beta_{00}+\beta_{11}\right)-2\beta_{01}=0$ for $\beta_{\left(2\right)}^{*}=0$ and its normal vector $a_{*}=\left(1,1,-2\right)$. This plane separates simulated data points based on their level of asymptotic cell segregation $\bar{\omega }$. This means, the direction $a_{*}$ indeed describes the direction of order progression, see color transition in Figure 5.

**Figure A1:**
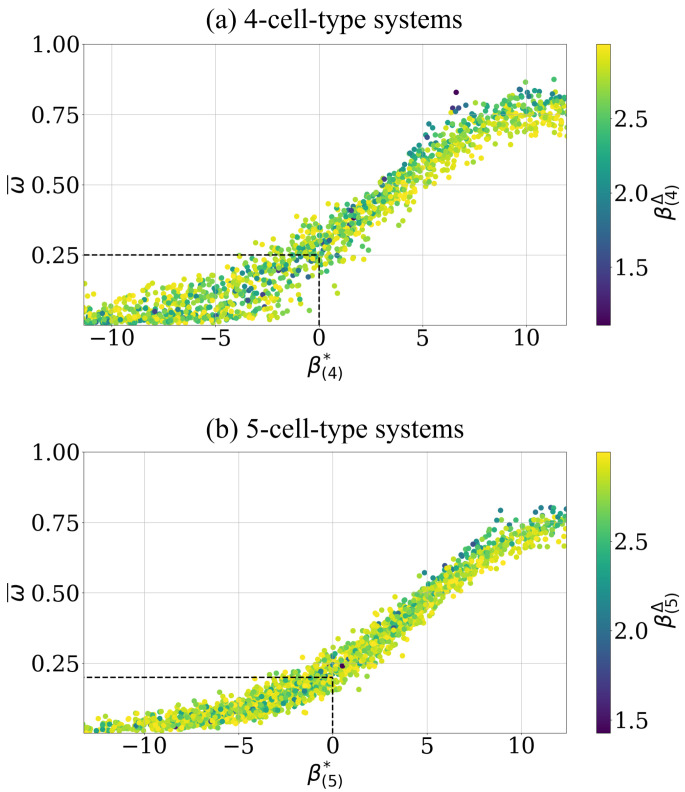
The level of the asymptotic cell segregation is predicted by the effective adhesion parameter for 4- and 5-cell-type systems. (**a**) Each point corresponds to a simulation whose intercellular adhesion parameter is drawn uniform, i.e., $\beta_{\left(4\right)}∼U{\left[-4,4\right]}^{10}$ with an additional condition such as $\beta_{\left(4\right)}^{\Delta }<3$, see Equation (17), to reduce scattering for visibility, see text of Figure 4 for details. The start configuration is random, i.e., initial order indicator ω(t=0)≈0.25. The curve $\omega (\beta_{\left(4\right)}^{*})$ intersects with the predicted point $\omega (\beta_{\left(4\right)}^{*}=0)=0.25$ (highlighted by black dashed lines). A data point represents the asymptotic estimate $\bar{\omega }$ of this simulation after ≈300000 cell switches, along with the corresponding effective adhesion parameter $\beta_{\left(4\right)}^{*}$ and convergence parameter $\beta_{\left(4\right)}^{\Delta }$ (color bar). (**b**) Analogous representation for the data points of 5-cell-type systems, starting with random configurations, i.e., initial order indicator values ω(t=0)≈0.20. The curve $\omega (\beta_{\left(5\right)}^{*})$ intersects with the predicted point $\omega (\beta_{\left(5\right)}^{*}=0)=0.20$ (highlighted by black dashed lines). The intercellular adhesion parameters are also drawn uniform, i.e., $\beta_{\left(5\right)}∼U{\left[-2.5,2.5\right]}^{15}$ with the additional condition $\beta_{\left(5\right)}^{\Delta }<3$.

We present analogous analysis for 4- and 5-cell-type systems, see Appendix C, Figure A1 and find a strong support of the analytically predicted effective adhesion parameter as well.

#### 3.3.2. Estimating the Effective Adhesion Parameter Using Statistical Learning Methods

We confirmed qualitatively the compliance between our prediction and the numerical data in the previous section. Now we confirm additionally our prediction by comparing the analytical effective adhesion parameters for two and three cell types to the corresponding estimates obtained via two statistical learning methods, Support Vector Machines (SVM) and Logistic Regression (Logit-Model) [16]. More precisely, for the general case of *N* cell types, the learning methods provide a hyperplane equation of the effective adhesion parameter $\beta_{\left(N\right)}^{*}$. Therefore, we quantify statistically the deviation from our analytical prediction. This is done for $\beta_{\left(2\right)}^{*}$ and $\beta_{\left(3\right)}^{*}$ based on the numerical data $\bar{\omega }\left(\beta \right)$ referred to in the previous section and displayed in Figure 4a,b, respectively. Both methods are suited for this task because they fit the linear dependency of the parameter $\beta^{*}$ on the intercellular adhesion parameters β, are well documented and directly available from libraries such as scikit-learn for Python [17], and, in contrast to some other approaches like deep learning, both quantify the factors that lead to classification.

The learning methods provide a hyperplane equation as a linear predictor to separate classified data points. Thus, the numerical data points $\bar{\omega }\left(\beta \right)$ are first classified based on their asymptotic order indicator $\bar{\omega }$ and a classification threshold. As thresholds, we chose the random configurations, i.e. $\bar{\omega }\geq 0.5$ for two cell types and $\bar{\omega }\geq 0.33$ for three cell types. Due to the symmetry of the effective adhesion parameter $\beta^{*}$, see Equations (7) and (13), the number of coefficients, which must be estimated, can be reduced to the constant a for the homotypic and the constant b for the heterotypic adhesion parameters. The resulting hyperplane equations for both 2-cell-type and 3-cell-type cases are presented in the headlines of Table 1a,b.

For the 2-cell-type case in Table 1a, both relative coefficients, for homotypic a/a≈1.00 and heterotypic b/a≈−1.99 intercellular adhesion parameters, are close to their predicted values 1 and −2, respectively. The relative intercept i/a, i.e. the value of $\beta_{\left(2\right)}^{*}$ which corresponds to the chosen threshold $\bar{\omega }=0.5$, is for both learning methods near zero, as predicted by our heuristic argument. These predictions of the SVM and the Logit-Model are of high quality as indicated by model accuracy values close to one. This quantitative result of the statistical learning methods in Table 1a support the analytical prediction for $\beta_{\left(2\right)}^{*}=\beta_{00}+\beta_{11}-2\beta_{01}$.

Analogous for three cell types, the results of Table 1b support the analytical results by estimating $\beta_{\left(3\right)}^{*}=\beta_{00}+\beta_{11}+\beta_{22}-\beta_{01}-\beta_{02}-\beta_{12}$ with convincing model accuracy of about 0.99 for both learning methods. The relative intercept i/a, i.e., the value of $\beta_{\left(3\right)}^{*}$ which corresponds to the chosen threshold $\bar{\omega }=0.33$, is for both learning methods close to zero, as predicted by our heuristic argument. We attribute the deviation of the relative intercept i/a from zero as well as the lower model accuracy compared to two cell types to insufficient convergence of the order indicators as predicted by the convergence speed $\beta_{\left(3\right)}^{\Delta }$.

We present analogous analysis for 4- and 5-cell-type systems, see Appendix C, Table A2 and again find a strong support of the analytically predicted parameters defining the effective adhesion parameter.

### 3.4. The Impact of Interfacial Tension, Adhesion or Repulsion on Cell Segregation

**Figure 6:**
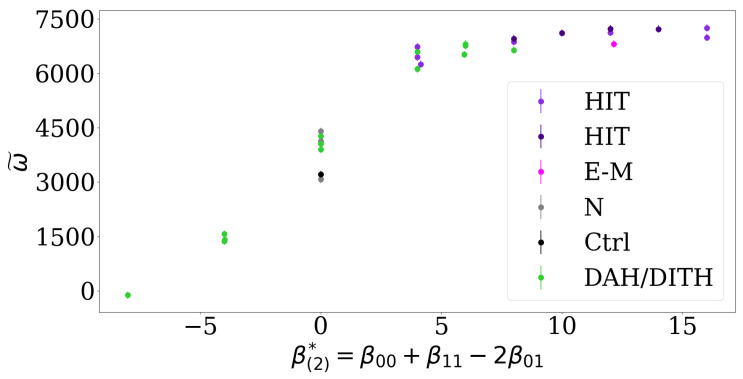
The effective adhesion parameter resolves previous discussions about the impact of interfacial tension compared to adhesion or repulsion. The indicator $\tilde{\omega }$ is analogous to the order indicator ω and is calculated by subtracting the length of heterotypic interfaces (LHI) reported in Canty et al. (2017) [11] from an upper bound of 8000 of all lattice site connections, the EAP is computed from the relative contact tensions $T_{ij}$ used there: $\beta_{\left(2\right)}^{*}=-T_{00}-T_{11}+2T_{01}=\beta_{00}+\beta_{11}-2\beta_{01}$. In contrast, Canty et al. [11] propose that the asymptotic level of segregation is determined by a discrete number of scenarios into which all relative contact energies can be sorted. These scenarios are: Differential Adhesion Hypothesis / Differential Interfacial Tension Hypothesis (DAH/DITH), control (Ctrl), two cases of High Heterotypic Interfacial Tension Hypothesis (HIT), ectoderm-mesoderm energies (E-M), negative control (N). The prediction in [11] was that HIT leads to the highest level of segregation, while E-M, N, Ctrl and DAH/DITH lead to lower segregation.

For two cell types, the EAP resolves previous discussions about the impact of interfacial tension on cell segregation compared to interfacial adhesion or repulsion [11]: We predict that a higher level of segregation is reached when the EAP is large, which can be achieved by both heterotypic repulsion or differential adhesion. In fact, transforming the relative contact tensions used in ref. [11] to the corresponding EAP by identifying high tensions $T_{ij}$ in the experiments and the Cellular Potts model (CPM) with low adhesion parameters $\beta_{ij}=-T_{ij}$ in the DMM, we can predict the experimental and numerical observations there, see Figure 6. In particular, we find that low lengths of heterotypic interfaces (LHI) are observed for high EAP values, analogous to the DMM. Figure 6 shows that the EAP is a better predictor for the asymptotic segregation behavior than the classification according to the DAH/DITH and HIT hypotheses.

Thus, we propose that rather than differentiating between intercellular adhesion and contact tension, the combined effect of intercellular contact on cellular motility, which is quantified by the EAP, should be focused on. Furthermore, the correct prediction of the asymptotic level of segregation in the CPM simulations of Ref. [11] using the effective adhesion parameter $\beta^{*}$ derived for the DMM suggests that our results may be directly applicable to CPM models of cell segregation. This is plausible due to the analogous structure of the exponent of the cell switch rates in the DMM and the energy functional in the CPM.

## 4. Discussion

We study analytically and numerically the differential migration model of Voss-Böhme and Deutsch (2010) [12], which is a cell-based model incorporating differential hypothesis [8], differential interfacial hypothesis [10], and High Heterotypic Interfacial Tension Hypothesis [11] within a unified framework. We generalize the existence and form of an effective adhesion parameter (EAP) guiding the asymptotic level of segregation, which is already known for systems with two cell types [12], to systems with an arbitrary number of cell types. We additionally predict the critical value of this effective adhesion parameter at which the system remains randomly mixed. For the case of two and three cell types, we confirm these theoretical predictions numerically and quantify the form of the effective adhesion parameter independently using statistical learning methods. The analogous results for 4- and 5-cell types suggest that our findings are valid in systems with even higher number of cell types. For two cell types, we show that the EAP resolves previous discussions about the impact of interfacial tension on cell segregation compared to interfacial adhesion or repulsion [11]. Thus, we propose that rather than differentiating between intercellular adhesion and contact tension, the combined effect of intercellular contact on cellular motility, which is quantified by the EAP, should be focused on.

Most previous studies focus on the simplest case of two cell types [7,13,14]. Although three and more cell types are more relevant in real biological systems, studies of cell segregation and pattern formation with three or more cell types are still rare [15]. By analytically and numerically demonstrating the existence of cell segregation for three cell types and characterizing its dynamics, we extent results from two cell types to the more general case.

The differential migration model has recently been applied successfully to reproduce experimental cell segregation data for two cell types [18]. In particular, the match between model and experimental observation demonstrated that contact tension and adhesion are sufficient to explain segregation data without additional mechanisms of collective motion. Analogously, our analytical and numerical results can be applied to cell segregation experiments with three or more cell types.

## Figures and Tables

**Table 1:** The effective adhesion parameter is confirmed by statistical learning methods. (**a**) For the estimation of the effective adhesion parameter $\beta_{\left(2\right)}^{*}$, 2000 simulations are conducted under the same conditions as described in Figure 4a. After simulation, each data point is classified: $\bar{\omega }>0.5$ as class 1, else class −1. The classified data points are used by the SVM and Logit-Model to estimate the homotypic a and the heterotypic b coefficient as well as the intercept i according to the table head. The model accuracy is tested via 5-fold cross-validation. (**b**) Analogous for the 3-cell-type systems the simulation conditions are described in Figure 4b. For data point classification applies: $\bar{\omega }>0.33$ as class 1, else class −1. For details on the estimation process as well as the model accuracy test, see Appendix E.

(a) 2-cell-type systems	$0=a\left(\beta_{00}+\beta_{11}\right)+b\beta_{01}+i$			Model
	a/a	b/a	i/a	Accuracy
SVM	1.0000	−1.9764	−0.0374	0.9965
Logit	1.0000	−1.9763	−0.0455	0.9965
Theoretical prediction	1	−2	0	
**(b) 3-cell-type** **systems**	$0=a\left(\beta_{00}+\beta_{11}+\beta_{22}\right)+b\left(\beta_{01}+\beta_{02}+\beta_{12}\right)+i$			**Model**
	a/a	b/a	i/a	**Accuracy**
SVM	1.0000	−0.9994	0.2835	0.9895
Logit	1.0000	−0.9935	0.2624	0.9900
Theoretical prediction	1	−1	0	

**Figure A2:**
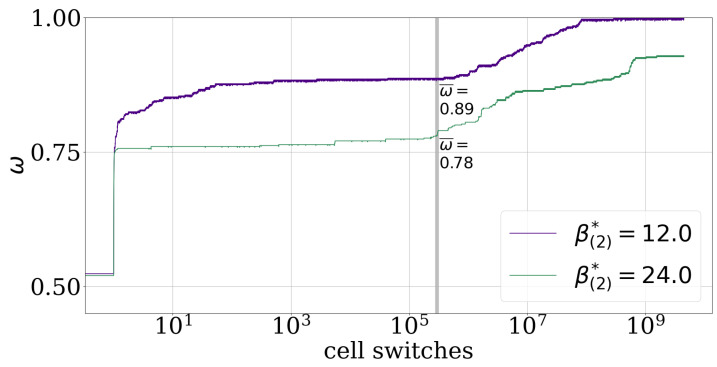
The effective adhesion parameter also influences the convergence speed exemplified with 2-cell-type systems. In the case of $\beta_{\left(2\right)}^{*}=12$ (violet) the intercellular adhesion parameters are (2.0,2.0,−4.0) and for $\beta_{\left(2\right)}^{*}=24$ (green) they are (4.0,4.0,−8.0). Both simulations have a random start configuration, i.e., initial order indicator ω(t=0)≈0.5 and run for about 7.2 million cell switches. The asymptotic estimate $\bar{\omega }$ for the last 10% of ≈300000 cell switches (gray area) is written on the corresponding time-series.

## Data Availability

The authors confirm that all data underlying the findings are fully available without restriction. All relevant data are contained within the manuscript and its appendix.

## Appendix A. PCA Implementation

The Differential Migration Model (DMM) [12] is implemented as a rule-based probabilistic cellular automaton (PCA). Instead of the classical discrete-time implementation via Monte-Carlo steps, a Gillespie-like algorithm is used which simulates the PCA in continuous time. For this, a support data structure is employed which tracks all heterotypic cell switches. These switches correspond to all possible events of the system for the corresponding lattice configuration η at a given point in time. The code effort is higher compared to more intuitive time-discrete algorithms due to the necessary update mechanics of the data structure but it allows the performance of one heterotypic cell switch per iteration step, since there are iteration steps without actual events in the analogous time-discrete Monte-Carlo simulation. At each iteration step, one entry from the support data structure is randomly chosen according to a weight which is calculated from the corresponding cell switch rate c(x,y,η), see Equation (2). Afterwards, the associated lattice sites x,y interchange their occupations. Then all entries of the support data structure that are affected by the configuration change are updated.

**Algorithm A1** The PCA Algorithm.

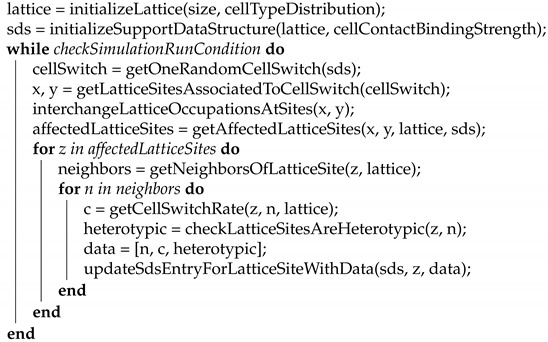

### Appendix A.1. initializeLattice

Initially, the square lattice *S* has the start configuration $\eta_{0}\in X$. The lattice *S* is described by $S={\left\{0,1,\cdots ,L\right\}}^{2}$ where L=25. The parameter called "cellTypeDistribution" determines the total amount of cells for each cell type w∈W within the lattice.

### Appendix A.2. initializeSupportDataStructure

The support data structure holds the information about the neighbors of all lattice sites together with the corresponding cell switch rates and whether a cell–cell contact is heterotypic or not. The parameter named "cellContactBindingStrength" provides the intercellular adhesion parameters $\beta_{\left(N\right)}$ to calculate the cell switch rate.

### Appendix A.3. checkSimulationRunCondition

To finalize a simulation a run condition is met. For this, either an iteration step count or a normalized order indicator value is used. In most cases, the simulation is stopped at 312,500 cell switches except for the data in Figure 3 where thresholds of the order indicator are used.

### Appendix A.4. getOneRandomCellSwitch

One heterotypic entry is drawn from the support data structure. This heterotypic cell switch identified by *k* has the corresponding cell switch rate $c^{\left(k\right)}\left(x,y,\eta \right)$. This rate belongs to the set ζ(η) of all heterotypic cell switch rates, given the lattice configuration η∈X. The probability $p^{\left(k\right)}$ that a cell switch *k* is chosen correlates directly with $c^{\left(k\right)}\left(x,y,\eta \right)$. That is shown in Equation (A1):

(A1) $$\begin{array}{c} \zeta (\eta ):=\{c(x,y,\eta )|x,y\in S,\;\eta (x)\neq \eta (y)\},\;\\ p^{\left(k\right)}:=\frac{c^{\left(k\right)}\left(x,y,\eta \right)}{\sum_{j=1}^{n}c^{\left(j\right)}\left(x,y,\eta \right)}\;\mathrm{with}\;n=\left|\zeta \left(\eta \right)\right|\;\mathrm{and}\;\sum_{j=1}^{n}p^{\left(j\right)}=1 \end{array}$$

### Appendix A.5. getLatticeSitesAssociatedToCellSwitch

The lattice sites x,y∈S from the chosen cell switch are obtained.

### Appendix A.6. interchangeLatticeOccupationsAtSites

The new occupation η(x)∈W of lattice site x∈S becomes the former occupation η(y)∈W of lattice site y∈S and vice versa, according to Equation (1).

### Appendix A.7. getAffectedLatticeSites

The set $Z_{\eta \to \eta_{xy}}$ of all lattice sites affected by the cell switch and the resulting configuration change $\eta \to \eta_{xy}$, see Equation (1), is composed of the direct neighbors u(x,y) of the lattice sites x,y∈S associated with the cell switch $\eta \to \eta_{xy}$ and their neighbors. Thus, it is

$$\begin{array}{c} Z_{\eta \to \eta_{xy}}:=\underset{e\in u(x,y)}{⋃}N\left(e\right)\;\mathrm{with}\;u\left(x,y\right):=N\left(x\right)\cup N\left(y\right). \end{array}$$

### Appendix A.8. getNeighborsOfLatticeSite

For a lattice site z∈S the neighborhood N(z) is obtained, see description to Equation (2).

### Appendix A.9. getCellSwitchRate

The cell switch rate c(x,y,η) between two lattice sites *x* and *y* is calculated according to Equation (2).

### Appendix A.10. checkLatticeSitesAreHeterotypic

Two lattice sites x∈S and y∈S are heterotypic if their corresponding cell types η(x),η(y) are not equal.

### Appendix A.11. updateSdsEntryForLatticeSiteWithData

The updated data in terms of cell switch rate and heterotypic cell contact of the neighborhood member n∈N(z) is assigned to the neighborhood host *z* within the support data structure.

## Appendix B. Order Indicator

**Table A1:** Highest and lowest number of homotypic connections for ideal 2- and 3-cell-type systems. These values are used to normalize the total sum of homotypic connections, see Equation (3).

	2-Cell-Type Systems	3-Cell-Type Systems
$d_{max}$	1200	1175
$d_{min}$	50	0

If we consider a lattice $S={\left\{0,1,\cdots ,L\right\}}^{2}$ then the ideal case of a fully sorted configuration consists of stripes for each of the *N* cell types, each with a height of $\frac{L}{N}$ and a length of *L*. In this scenario the highest amount $d_{max}$ of homotypic lattice site connections is $d_{max}=2\cdot L^{2}-n\cdot N$, since every lattice site has two unique homotypic connections except for those at cell-type interfaces. These only have one homotypic connection. For the same ideal scenario, a chessboard configuration where no homotypic connections exist can be achieved for the right lattice side length *L*. Therefore, $d_{min}=0$.

In practice, a discrete lattice with $25^{2}$ lattice sites cannot be separated into even stripes. Due to this imperfection, small deviations from these values occur in data. In the case of 2-cell-type systems, a lattice side of L=25 causes $d_{min}=2\cdot L=50$ because every cortical lattice site has one homotypic connection due to periodicity.

In other publications such as Canty et al. (2017) [11], the level of cell segregation and clustering is quantified by the length of the heterotypic interface, here we use the amount of homotypic lattice site connections, see Equation (3).

## Appendix C. Numerical Evidence for the Effective Adhesion Parameter with Extended Cell-Type Number

**Table A2:** The effective adhesion parameter is confirmed by statistical learning methods for 4 and 5 cell types. (**a**) For the estimation of the effective adhesion parameter $\beta_{\left(4\right)}^{*}$, 2000 simulations are conducted under the same conditions as described in Figure A1a. After simulation, each data point is classified: $\bar{\omega }>0.25$ as class 1, else class −1. The classified data points are used by the SVM and Logit-Model to estimate the homotypic a and the heterotypic b coefficient as well as the intercept i according to the table head. The model accuracy is tested via 5-fold cross-validation. (**b**) Analogous for the 5-cell-type systems the simulation conditions are described in Figure A1b. For data point classification applies: $\bar{\omega }>0.20$ as class 1, else class −1. For details on the estimation process as well as the model accuracy test, see Appendix E.

(a) 4-cell-type systems	$0=a\sum_{i=0}^{3}\beta_{\mathrm{ii}}+b\sum_{i<j}^{3}\beta_{\mathrm{ij}}+i$			Model
	a/a	b/a	i/a	Accuracy
SVM	1.0000	−0.6553	0.8022	0.9615
Logit	1.0000	−0.6625	0.7330	0.9610
Theoretical prediction	1	−2/3	0	
**(b) 5-cell-type** **systems**	$0=a\sum_{i=0}^{4}\beta_{\mathrm{ii}}+b\sum_{i<j}^{4}\beta_{\mathrm{ij}}+i$			**Model**
	a/a	b/a	i/a	**Accuracy**
SVM	1.0000	−0.5030	0.5889	0.9555
Logit	1.0000	−0.4913	0.5992	0.9565
Theoretical prediction	1	−0.5	0	

## Appendix D. The Impact of the Effective Adhesion Parameter on the Convergence Speed

Figure A2 shows that the larger the EAP $\beta^{*}$ is the more cell switches are needed before a simulation reaches its asymptotic segregation state which is a fully sorted pattern in the case of $\beta_{\left(2\right)}^{*}=12$ and $\beta_{\left(2\right)}^{*}=24$ according to the DMM, see Section 2.1. This effect is visible from about $\beta^{*}>4$ and the cause is still unknown.

## Appendix E. Statistical Learning Methods

All methods, SVM and Logistic regression as well as the 5-fold cross-validation, are executed in default mode and as recommended by the library scikit-learn. This includes among others the hyperparameter C=1.0. The smaller this inverse regularization parameter is the more regularization is applied and the lower is the risk of model over fitting. This also can increase the numerical stability. The default penalization is in both cases l2-penalty [17]. Since all intercellular adhesion parameters are from the same distribution, see caption of Figure 4, preprocessing steps such as data scaling are omitted. Other execution details are:

- SVM: used algorithm implementation “SVC”
- SVM: kernel “linear”
- Logit: used training algorithm/solver “lbfgs”
